# Host phenology‐driven infestation patterns of the carob moth ( *Ectomyelois ceratoniae* ) in Mediterranean walnut orchards: insights from comparison with codling moth ( *Cydia pomonella* )

**DOI:** 10.1002/ps.70767

**Published:** 2026-04-01

**Authors:** Fortuna Miele, Flavia de Benedetta, Enza Petito, Giovanna Avventura, Francesco Migliaccio, Francesco Nugnes, Umberto Bernardo

**Affiliations:** ^1^ Institute for Sustainable Plant Protection – National Research Council (IPSP‐CNR) Portici Italy

**Keywords:** carob moth, codling moth, early harvest, packing tissue brown stage, phenology‐based IPM, pheromone trapping

## Abstract

**BACKGROUND:**

While *Cydia pomonella* has long been regarded as the key lepidopteran pest of walnut in Europe, field observations increasingly indicate that *Ectomyelois ceratoniae* is becoming dominant in Mediterranean orchards. To assess their relative importance, we compared the incidence and seasonal dynamics of both species across multiple orchards and years. Because fruit susceptibility varies with ripening stage, aligning pest monitoring with host phenology may improve the timing and integration of control tactics. This study examined the synchrony between walnut phenology and the temporal patterns of adult occurrence and larval infestation of *E. ceratoniae* and *C. pomonella* to develop phenology‐driven integrated pest management (IPM) strategies.

**RESULTS:**

Over two seasons and three orchards, *E. ceratoniae* consistently outnumbered *C. pomonella* in trap captures and larval recoveries. Infestation was concentrated at advanced ripening stages, peaking between the packing tissue brown (PTB) stage and husk dehiscence. Adult captures systematically preceded larval entry into the kernels, confirming the effectiveness of pheromone traps as early‐warning tools when interpreted alongside phenological observations. The stabilized lure (7*Z*,9*E*,11‐dodecatrienyl formate with butylated hydroxytoluene) produced reliable flight curves. The interval between pistillate flower receptivity and PTB was highly consistent (≈126–129 days), allowing reliable estimation of the high‐risk period.

**CONCLUSION:**

*Ectomyelois ceratoniae* was responsible for most of the infestation pressure in ripening and mature fruits, supporting its role as the main late‐season lepidopteran pest of Mediterranean walnuts. Early harvest after PTB stage, combined with orchard sanitation and pheromone‐based monitoring, can substantially reduce infestation risk. These findings provide a practical, phenology‐driven IPM framework to safeguard walnut quality and support the inclusion of *E. ceratoniae* in official production guidelines. © 2026 The Author(s). *Pest Management Science* published by John Wiley & Sons Ltd on behalf of Society of Chemical Industry.

## Graphical abstract

In Mediterranean walnut orchards, *Ectomyelois ceratoniae* replaces *Cydia pomonella* as the primary pest. Capture peaks between packing tissue brown (PTB) stage and husk dehiscence define a critical window for monitoring and early harvest to prevent kernel infestation.

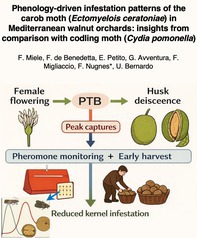

## 1 INTRODUCTION

In recent decades, changes in climate, global trade, and pest management practices have promoted the emergence or resurgence of insect pests that were previously considered of secondary importance.^1^, ^2^, ^3^, ^4^ Among these, the carob moth *Ectomyelois ceratoniae* (Zeller) (Lepidoptera: Pyralidae) has become a growing threat to walnut (*Juglans regia* L.) in the Mediterranean basin. This moth is cosmopolitan and highly polyphagous, with records on more than 43 host species across 18 plant families, including walnut, almond, citrus, pomegranate, and date palm.^5^, ^6^, ^7^ Although regarded as endemic to Mediterranean agroecosystems (but see Miele *et al*.^7^), its impact on walnut has only recently attracted attention, with outbreaks reported both in orchards and in storage facilities.^8^, ^9^, ^10^

Population dynamics vary with hosts and environments. In citrus orchards and pomegranate, *E. ceratoniae* completes four to five generations per year, with the most damaging coinciding with fruit maturity. Late‐season larvae enter diapause inside fruits and overwinter. Diapause is triggered by short photoperiods and low temperatures, while pupation and adult emergence resume in spring under longer days and warmer conditions. This strategy allows overwintering larvae to act as a reservoir for population resurgence.^11^, ^12^ Overwintering studies further show that larvae persist inside fruits with limited mortality, suggesting that cold does not substantially constrain populations and may allow rapid resurgence in spring.^13^

This combination of frequent outbreaks and strong overwintering ability highlights the ecological flexibility of *E. ceratoniae*. The species is particularly problematic in Europe, where stringent quality standards impose zero tolerance for live insects in kernels.^14^ This standard provides a regulatory framework for walnut quality but was not directly evaluated in the present study.

Across crops, fruit phenology consistently shapes infestation patterns. In pomegranate, cracked fruits were attacked at rates 5–15 times higher than intact fruits early in the season. Later intact fruits became key refuges for overwintering larvae, with survival more than three‐fold higher than in cracked fruits.^15^ Almonds show a parallel mechanism: *E. ceratoniae* oviposits mainly at hull split, when openings provide access to the kernel, and develops in overwintering ‘mummy’ nuts.^16^ Feeding on maturing fruits also promotes fungal infection and premature yellowing, indicating that tissue weakening at advanced phenological stages enhances susceptibility.^16^, ^17^ Field experiments with traps baited with pomegranate confirmed this pattern: cracked fruits attracted significantly more females than intact fruits, underscoring the role of phenology in host‐finding and oviposition.^18^

Population growth depends on both host and fruit stage. In pomegranate, demographic rates peaked at intermediate stages while very young or fully ripe fruits were less suitable.^17^, ^19^ Under controlled conditions, pomegranate and pistachio supported the highest reproductive potential, whereas fig and date were much less suitable.^20^ On date palm, larvae performed better on early than on late fruits.^21^ A recent study on pistachio also showed that host plant traits influence not only *E. ceratoniae* demography but also parasitoid effectiveness, with cascading effects across trophic levels.^22^ Rising temperatures may exacerbate these dynamics by accelerating development, shortening generation times, and increasing the number of annual cycles.^23^, ^24^ Where hosts are continuously available, this can result in overlapping generations and sustained late‐season infestations. The challenge is particularly severe in walnut, where late fruit ripening coincides with peak pest pressure. Nevertheless, Italian integrated production guidelines still focus primarily on *Cydia pomonella* (L.) (Lepidoptera: Tortricidae) and do not include *E. ceratoniae*, suggesting that its impact may be underestimated in Mediterranean and European walnut production systems.

Taken together, these observations point to the central role of walnut phenology in shaping vulnerability to carob moth damage. Phenology‐based monitoring can therefore provide reliable indicators of high‐risk periods, helping to optimize the timing of interventions within integrated pest management (IPM) strategies.^25^

This study explores the synchrony between walnut ripening and *E. ceratoniae* infestation across multiple orchards and seasons. Specifically, we (i) determined the timing of key phenological stages, (ii) quantified larval infestation at each host phenological stage, and (iii) evaluated the implications for predicting and mitigating late‐season infestation risk. By integrating pest biology with host phenology, this work offers a framework for phenology‐driven IPM strategies against *E. ceratoniae* in walnut production systems. The study focuses on phenology‐driven infestation patterns and risk windows rather than on modelling population growth or long‐term abundance trends.

## 2 MATERIALS AND METHODS

### 2.1 Location and plant materials

The experimental trial was conducted in 2023 and 2024 in three walnut orchards located in Campania (southern Italy). The cultivar was ‘Sorrento’, and trees were spaced 10 m apart both between and within rows. Trees were trained to a high‐trunk open vase with four main branches. At Campo Base, the walnut orchard was intercropped with citrus trees, whereas at Mandrile it consisted of a specialized walnut orchard, and at Erasmo it was intercropped with hazelnut trees (Table 1). Pest management regimes were uniform across all orchards, and no chemical control measures targeting walnut pests were applied during the study period. No weed control or pruning was performed in any orchard.

**Table 1:** Description of the three walnut orchards where the trial was carried out

Site	Location	Coordinates	Size (ha)	Orchard age (year)	Orchard composition
Campo Base	Palma Campania (Naples)	40° 51′ 6.62″ N	2.5	**≈** 30	*Juglans regia*, *Citrus* spp.
		14° 33′ 27.40″ E			
Mandrile	Palma Campania (Naples)	40° 50′ 55.25″ N	2.7	**≈** 30	*Juglans regia*
		14° 31′ 49.80″ E			
Erasmo	Nola (Naples)	40° 52′ 52.82″ N	1.3	**≈** 30	*Juglans regia*, *Corylus avellana*
		14° 31′ 20.68″ E			

### 2.2 Walnut phenology and lepidoptera damage assessment

During the 2023–2024 seasons, field surveys and fruit samplings were performed to document walnut phenological stages and assess lepidopteran damage.

Phenological observations focused on pistillate flower receptivity, the packing tissue brown (PTB) stage, and husk dehiscence. Pistillate flower receptivity was monitored as a key phenological stage and used as a temporal reference for subsequent stages of fruit development.^26^, ^27^ The PTB stage was included as a phenological indicator of kernel physiological ripening; in walnut, this stage corresponds to the time when the packing tissue between and around the kernel halves just turns brown and is commonly used to determine the optimal harvest time.^28^ Husk dehiscence, defined as the natural splitting and detachment of the husk from the shell, is considered a marker of external fruit ripeness. In walnut, this process signals hull maturity, which may occur after kernel ripening, thereby influencing optimal harvest timing and final nut quality.^29^ Phenological stages were recorded according to the BBCH scale (Biologische Bundesanstalt, Bundessortenamt and CHemical Industry), a standardized system that describes plant development using numerical codes.^30^ Female flower receptivity (BBCH 610) was monitored in all orchards through weekly visual observations starting in March of each year.^27^ Fruit development was subsequently followed until full external husk size (BBCH 79); at which stage walnut fruits were sampled to assess the PTB stage, hull dehiscence, and lepidopteran damage. In each walnut orchard, fruits were randomly sampled weekly from six trees located at least 50 m apart. From each tree, a total of five fruits were collected at each sampling date, selecting fruits from different canopy quadrants (north, east, south, and west) at approximately 2.5 m height, to ensure spatially representative sampling within the canopy. Sampled fruits were placed in double plastic bags, labelled, transported to the laboratory, and stored at 4 °C until analysis. In total, 30 fruits per orchard were collected at each sampling date. Over two seasons and three orchards, this resulted in 3330 sampled fruits. Fruits were analysed for both phenological development and pest damage. Every sampled fruit was first inspected externally for the presence of holes and larvae. Fruit development was then monitored, and the percentage of fruits that reached the hull‐breaking stage was recorded. Physiological maturity was assessed by examining walnut halves and determining the proportion of nuts that had reached the PTB stage. At each sampling date, insect presence and kernel infestation were also recorded. Lepidopteran larvae were collected, counted, and preserved in labelled Eppendorf tubes containing 99% ethanol, and stored at −20 °C, until morphological and molecular identification. Infestation was expressed as the presence or absence of larvae within sampled kernels and summarized as seasonal incidence. Larval abundance data were subsequently aggregated as cumulative counts per orchard and season for statistical comparisons and no quantitative assessment of yield loss or kernel quality degradation was performed.

### 2.3 Lepidoptera monitoring activities with traps

Traps for insect monitoring were placed at the beginning of March in both 2023 and 2024, when the plants were in dormant bud stage (00‐BBCH). In each site, six delta sticky traps (Biogard division, CBC (Europe) Srl, Grassobbio (BG), Italy) were placed 100–200 m apart: three traps were baited with the *E. ceratoniae* lure (7*Z*,9*E*,11‐dodecatrienyl formate + butylated hydroxytoluene) and three with the *C. pomonella* lure ((*E*,*E*)‐8,10‐dodecadienol). In preliminary tests conducted in 2022, commercially available lures lacking the stabilizer butylated hydroxytoluene were ineffective, whereas the stabilized formulation consistently attracted males and was therefore used in this study.

Traps were checked weekly until no fruits remained on the ground, and pheromones were replaced monthly. Captured specimens were morphologically identified.

### 2.4 Morphological and molecular identification of insects

Both larvae and adults were examined under a Leica M165C auto montage microscope (Leica Microsystems, Mannheim, Germany). Larvae specimens were identified using available taxonomic keys.^31^, ^32^ Although the lures are designed to be species‐specific, morphologically similar non‐target species were often captured, particularly in *E. ceratoniae* traps. Therefore, all doubtful *E. ceratoniae* specimens were examined through genitalia‐based morphological identification using temporary microscope slides.^33^

In addition, molecular identification was carried out on several specimens following the protocol described in Miele *et al*.^7^.

### 2.5 Statistical analysis

Statistical analyses were designed to compare species‐specific levels of adult activity and larval infestation across phenological periods and seasons, rather than to model population growth or infer temporal trends. Larval infestation was assessed using cumulative larval counts aggregated by orchard and year; no time‐standardized infestation indices (e.g., larvae per fruit‐day or insect/trap/day) were calculated.

All analyses were conducted within a unified framework, with site and year included as grouping (blocking) factors to control for orchard‐level variability.

For each site and year, total adult catches and total larval counts were compared between the two species. Because only two species were compared, pairwise differences were directly assessed within each model, and *post hoc* testing was not required.

Assumptions of normality and homoscedasticity were verified prior to analysis. When assumptions were met, one‐way analysis of variance (ANOVA) was applied; otherwise, data were transformed as appropriate, and if assumptions remained unmet, the Mann–Whitney *U* test was used.

To test for species‐specific differences in adult captures and larval infestation across phenological periods (pre‐fruit enlargement (1), fruit enlargement (2), PTB (3), and post‐PTB (4)), generalized estimating equations (GEEs) were applied with species as a categorical fixed factor. A Poisson distribution, exchangeable correlation structure, and robust variance estimator were specified to account for repeated measures from the same traps (or trees). Sampling date was included only to model within‐unit correlation and not interpreted as a biological predictor. Time was treated implicitly through phenological periods reflecting discrete biological phases of host susceptibility rather than as a continuous variable. This choice avoids fitting artificial temporal trends to zero‐inflated early‐season data and focuses the analysis on biologically meaningful interactions between pest activity and walnut development. Accordingly, no explicit species × time interaction based on sampling date was fitted; instead, species effects were evaluated across phenological periods. Larval infestation data were analysed separately from adult captures due to their discontinuous temporal distribution and frequent absence during early phenological stages (particularly for *E. ceratoniae*). For this reason, infestation was aggregated within phenological periods rather than modelled as a continuous temporal response variable. Weekly infestation percentages were not modelled over time due to the predominance of zero values during early phenological stages and the abrupt onset of larval infestation at later stages, which would result in zero‐inflated and poorly informative temporal trends. Formal correlation analyses between adult captures and larval infestation were therefore not performed, as the two variables are temporally decoupled, with extended intervals between initial adult activity and the onset of larval infestation, limiting the biological interpretability of direct statistical associations.

When the GEE model did not provide a satisfactory fit, as indicated by lack of convergence or unsatisfactory diagnostic evaluation, an alternative analysis was explicitly applied: data were analysed separately for each phenological period by aggregating total captures or infestations, and species‐specific differences were tested using one‐way ANOVA after verification of assumptions, or the Mann–Whitney *U* test when assumptions were not met.

All analyses were conducted using SPSS 29.^34^ Weekly sampling dates were assigned to phenological periods based on walnut developmental stage rather than calendar time. Each phenological period therefore comprised multiple consecutive sampling dates, with the number of observations per period varying among sites and years. Sampling effort was not strictly balanced across periods, and this temporal structure was accounted for within the GEE framework.

## 3 RESULTS

### 3.1 Walnut phenology

The timing of pistillate flower receptivity varied among sites and years, as reported in Table 2. Husk dehiscence began during the first week of September in both years and across all monitored orchards.

**Table 2:** Dates of pistillate flower receptivity and of the packing tissue brown (PTB) stage (50% fruits) recorded in the three orchards during the 2023 and 2024 seasons

Site	Receptivity 2023	Receptivity 2024	PTB 2023	PTB 2024
Campo Base	10 May	21 April	14 September	26 August
Mandrile	5 May	20 April	11 September	26 August
Erasmo	15 May	30 April	18 September	3 September

The PTB stage, reached by 50% of the sampled fruits, occurred between late August and mid‐September, with earlier occurrence in 2024 compared to 2023 (Table 2). The interval between pistillate flower receptivity and the PTB stage was highly consistent, ranging from 126 to 129 days across orchards and years in both 2023 and 2024.

### 3.2 Lepidoptera infestation and kernel infestation incidence

Almost all collected larvae belonged to two species, *E. ceratoniae* and *C. pomonella*, with 143 individuals recovered in 2023 and 100 in 2024, whereas a few specimens of *Cadra cautella* (Lepidoptera, Pyralidae) were also detected in the nuts and excluded from statistical analyses. Other Lepidoptera species were detected only sporadically and collectively accounted for approximately 0.81% of the total larvae recovered across all sites and years.

At Campo Base, *E. ceratoniae* represented 92.4% of the total larvae in 2023 and 83.3% in 2024. No citrus fruits were ever found to be infested by *E. ceratoniae* in this orchard. At Mandrile, *E. ceratoniae* accounted for 81.2% in 2023 and 80.9% in 2024. At Erasmo, *E. ceratoniae* comprised 35.1% of the larvae in 2023 and 86.5% in 2024.

Comparisons based on cumulative counts of *E. ceratoniae* and *C. pomonella* larvae highlighted significant differences among orchards and years (Table 3).

**Table 3:** Results of one‐way analysis of variance (ANOVA) and Mann–Whitney *U* tests comparing cumulative seasonal larval counts of *Ectomyelois ceratoniae* (EC) and *Cydia pomonella* (CP) recovered from walnuts across orchards and years

Site	Year	df	*F*	*P*	Species	Mean ± standard error
Erasmo	2023	1	0.914	0.393	EC	4.33 ± 0.88
					CP	7.00 ± 2.65
	2024	1	3.971	0.046	EC	10.67 ± 3.29
					CP	1.33 ± 0.33
Campo Base	2023	1	3.971	0.046	EC	25.00 ± 3.05
					CP	2.00 ± 1.00
	2024	1	3.232	0.072	EC	8.67 ± 3.48
					CP	1.67 ± 0.67
Mandrile	2023	1	4.091	0.043	EC	13.33 ± 0.67
					CP	2.33 ± 0.67
	2024	1	4.355	0.037	EC	5.33 ± 1.85
					CP	1.00 ± 0.00

**Figure 1:**
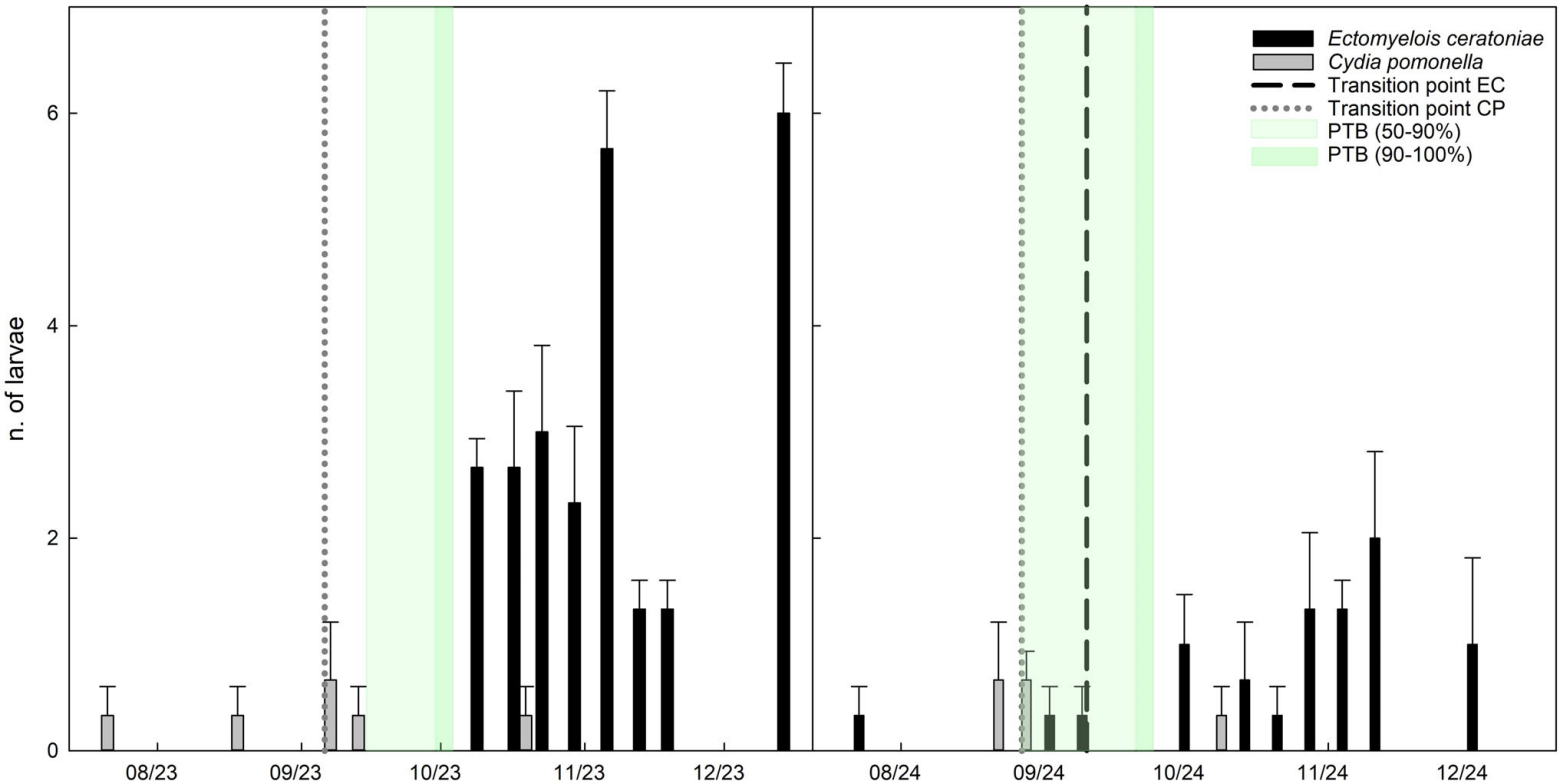
Temporal distribution of larvae of *Ectomyelois ceratoniae* (EC) and *Cydia pomonella* (CP) recovered from walnuts at Campo Base in 2023 and 2024, sampled at full external husk size. Bars represent mean ± standard error. The *y*‐axis represents the total number of larvae recovered per sampling date, aggregated across all sampled fruits within each orchard. Light and dark green shaded areas represent the PTB stages (50–90% and 90–100%). Vertical dashed lines indicate the transition point, that is, the shift from larvae being present only in the husk to their first detection inside the kernel. Absence of a vertical dashed line indicates direct development in the kernel.

**Figure 2:**
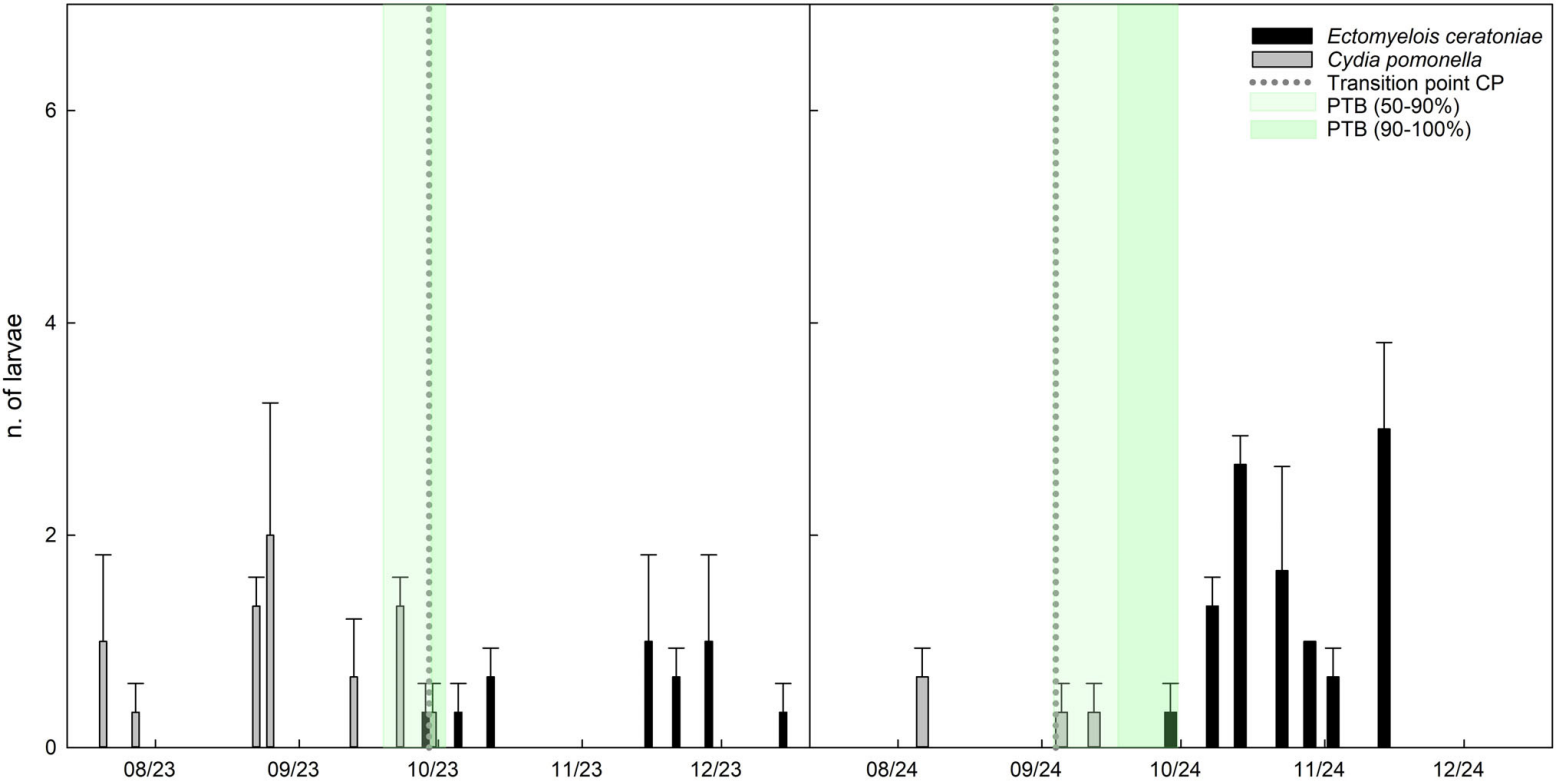
Temporal distribution of *Ectomyelois ceratoniae* (EC) and *Cydia pomonella* (CP) larvae recovered from walnuts at Erasmo in 2023 and 2024, sampled at full external husk size. Bars represent mean ± standard error. The *y*‐axis represents the total number of larvae recovered per sampling date, aggregated across all sampled fruits within each orchard. Light and dark green shaded areas represent the PTB stages (50–90% and 90–100%). Vertical dashed lines indicate the transition point, that is, the shift from larvae being present only in the husk to their first detection inside the kernel. Absence of a vertical dashed line indicates direct development in the kernel.

**Figure 3:**
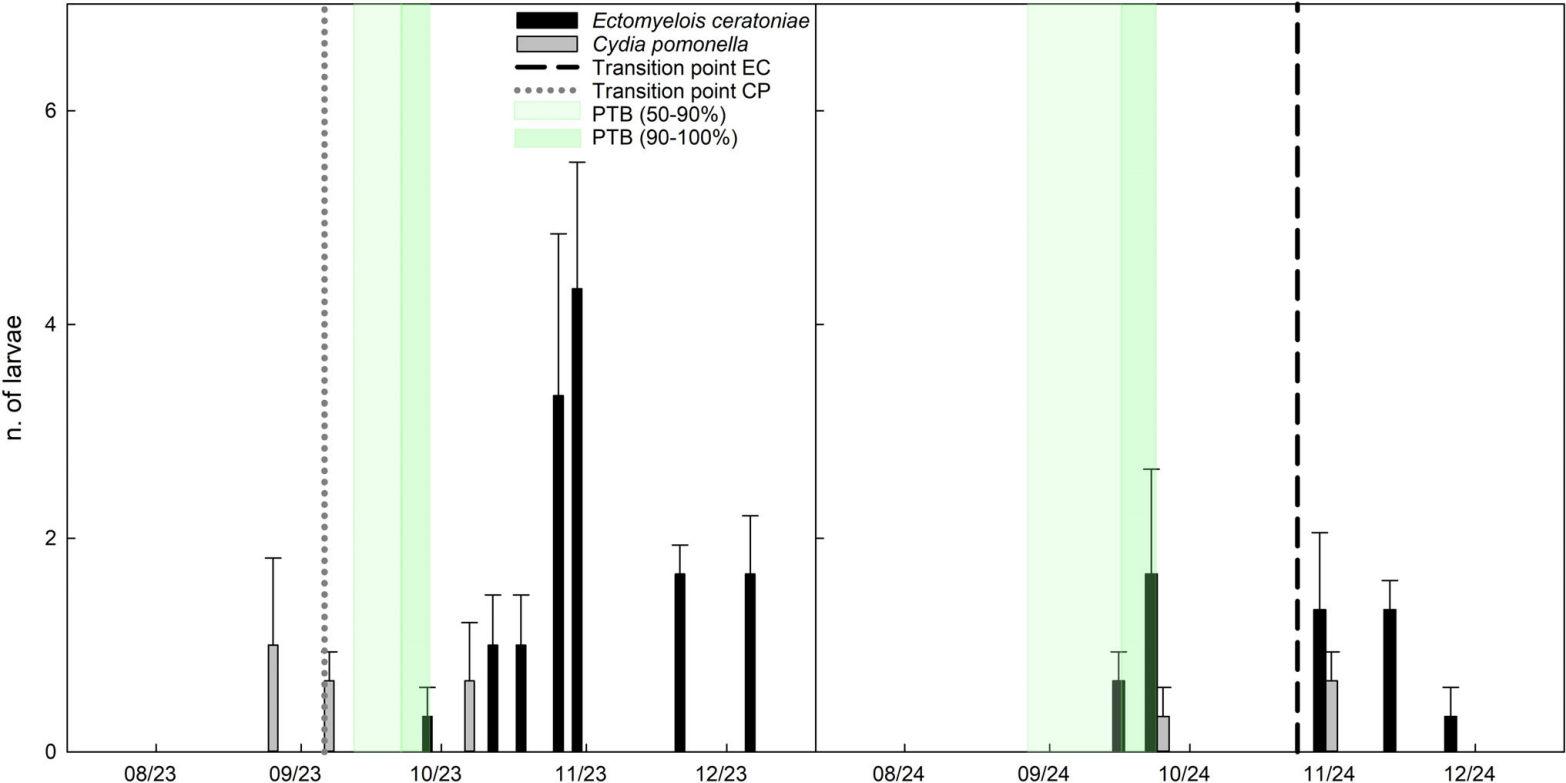
Temporal distribution of *Ectomyelois ceratoniae* (EC) and *Cydia pomonella* (CP) larvae recovered from walnuts at Mandrile in 2023 and 2024, sampled at full external husk size. Bars represent mean ± standard error. The *y*‐axis represents the total number of larvae recovered per sampling date, aggregated across all sampled fruits within each orchard. Light and dark green shaded areas represent the PTB stages (50–90% and 90–100%). Vertical dashed lines indicate the transition point, that is, the shift from larvae being present only in the husk to their first detection inside the kernel. Absence of a vertical dashed line indicates direct development in the kernel.

Table 3 summarizes overall species‐level differences in cumulative larval infestation at the orchard and year scale, providing a synthetic comparison of total larval counts. Seasonal variation across phenological periods is reported in Tables 4 and 5, while Figs 1, 2, 3 provide a descriptive visualization of larval occurrence throughout the season.

**Table 4:** Results of generalized estimating equation (GEE) models testing the effect of species on trap captures across phenological periods, orchard sites, and years

Site	Year	Period	*χ* ^2^	df	*P*	Species	Mean ± standard error
Campo Base	2023	1	0.905	1	0.341	EC	0.33 ± 0.33
						CP	1.00 ± 1.00
		2	34.652	1	<0.001	EC	36.67 ± 12.81
						CP	3.67 ± 1.20
		3	23.949	1	<0.001	EC	23.67 ± 7.31
						CP	2.67 ± 1.20
	2024	2	1.550	1	0.213	EC	10.00 ± 3.79
						CP	17.00 ± 6.11
		3	22.486	1	<0.001	EC	25.00 ± 11.27
						CP	4.58 ± 0.58
		4	52.992	1	<0.001	EC	46.67 ± 15.32
						CP	1.33 ± 0.67
Erasmo	2023	2	9.565	1	0.002	EC	8.33 ± 1.45
						CP	24.33 ± 10.17
		3	201.326	1	<0.001	EC	3.33 ± 1.76
						CP	1.33 ± 0.33
	2024	2	9.565	1	0.002	EC	2.67 ± 1.45
						CP	13.00 ± 4.04
		3	201.326	1	<0.001	EC	25.67 ± 3.84
						CP	2.33 ± 0.33
Mandrile	2023	2	128.881	1	<0.001	EC	23.67 ± 4.41
						CP	3.33 ± 0.33
		3	28.827	1	<0.001	EC	6.33 ± 2.67
						CP	1.00 ± 0.00
	2024	2	0.198	1	0.657	EC	6.67 ± 2.03
						CP	5.33 ± 2.85
		3	0.836	1	0.361	EC	7.67 ± 7.17
						CP	3.33 ± 2.03
		4	0.314	1	0.575	EC	1.33 ± 0.33
						CP	1.00 ± 0.58

*Note*: Models were fitted with a Poisson distribution, exchangeable correlation structure, and robust variance estimator. Period definitions: Period 1 – fruit enlargement to packing tissue brown (PTB) stage 50%; Period 2 – PTB stage 50–90%; Period 3 – PTB stage 90–100%; Period 4 – post‐PTB stage. Periods in which model convergence was not achieved are not shown.

**Table 5:** Results of Mann–Whitney *U* tests comparing total trap captures of *Ectomyelois ceratoniae* (EC) and *Cydia pomonella* (CP) within each phenological period, by site and year

Site	Year	Period	*H*	df	*P*	Species	Mean ± standard error
Campo Base	2023	4	4.355	1	0.037	EC	44.67 ± 10.27
						CP	0
	2024	1	1.000	1	0.317	EC	0
						CP	0.67 ± 0.67
Erasmo	2023	1	2.400	1	0.121	EC	0
						CP	2.00 ± 1.53
		4	2.634	1	0.105	EC	3.00 ± 1.53
						CP	0.67 ± 0.33
	2024	1	4.355	1	0.037	EC	0
						CP	5.33 ± 1.20
		4	4.355	1	0.037	EC	39.00 ± 10.02
						CP	0
Mandrile	2023	1	1.000	1	0.317	EC	0
						CP	0.33 ± 0.33
		4	4.355	1	0.037	EC	27.00 ± 6.43
						CP	0
	2024	1	0.000	1	1.000	EC	0
						CP	0

*Note*: The test was applied in periods where generalized estimating equation (GEE) model convergence was not achieved or where assumptions for parametric analysis were not met. Period definitions: Period 1 – fruit enlargement to packing tissue brown (PTB) stage 50%; Period 2 – PTB stage 50–90%; Period 3 – PTB stage 90–100%; Period 4 – post‐PTB.

Seasonal patterns of larval occurrence for both species are descriptively illustrated for Campo Base, Erasmo, and Mandrile (Figs 1, 2, 3). Each figure includes the estimated transition point for both species, defined as the shift from larvae being detected only in the husk to their first detection inside the walnut kernel. When larvae were never observed in the husk, it is assumed that they developed directly inside the kernel without an intermediate husk phase.

Some biological differences were observed from larval monitoring. Infestation by *C. pomonella* larvae was generally detected earlier than that of *E. ceratoniae*, with only two exceptions involving a few carob moth individuals (Campo Base and Mandrile 2024). Across sites and years, first detection of *C. pomonella* larvae preceded that of *E. ceratoniae* by approximately 41.83 ± 11.43 days, depending on orchard and season (Figs 1, 2, 3).

With a single exception (Mandrile 2024), codling moth larvae were first detected in the husk and later in the kernel, indicating a clear transition point. In contrast, *E. ceratoniae* larvae were often detected directly in the kernel and only rarely in the husk (Figs 1, 2, 3).

### 3.3 Lepidoptera adult monitoring with traps

Specimens of other lepidopteran species occasionally caught in the traps (*Anarsia lineatella* (Zeller) (Lepidoptera, Gelechiidae) and *Grapholita molesta* (Busck) (Lepidoptera, Tortricidae)) were excluded from the analysis and all other non‐target Lepidoptera were not systematically collected.

At Campo Base, *E. ceratoniae* accounted for 93.5% of total captures in 2023 and 78% in 2024. At Mandrile, the proportion of *E. ceratoniae* was 86.35% in 2023 and 59% in 2024. At Erasmo, *E. ceratoniae* represented 34.1% of captures in 2023 and 76.5% in 2024. Results of the statistical comparisons between trap captures of the two species are presented in Table 6.

**Table 6:** Results of one‐way analysis of variance (ANOVA) (first four analyses) and Mann–Whitney *U* tests (last two analyses) comparing cumulative seasonal trap captures of *Ectomyelois ceratoniae* (EC) and *Cydia pomonella* (CP) across sites and years

Site	Year	df	*F*	*P*	Species	Mean ± standard error
Campo Base	2023	1	29.396	0.006	EC	105.33 ± 27.23
					CP	7.33 ± 2.33
Erasmo	2023	1	1.674	0.265	EC	14.67 ± 2.85
					CP	28.33 ± 10.17
Erasmo	2024	1	11.061	0.029	EC	67.33 ± 13.12
					CP	20.67 ± 4.98
Mandrile	2024	1	0.370	0.576	EC	15.67 ± 9.21
					CP	9.67 ± 3.53

Site	Year	df	*H*	*P*	Species	Mean ± standard error
Campo Base	2024	1	3.971	0.046	EC	105.33 ± 27.23
					CP	7.33 ± 2.33
Mandrile	2023	1	3.971	0.046	EC	57.00 ± 11.60
					CP	4.67 ± 0.33

Results reported in Tables 4 and 5 show that capture differences were generally not significant in Period 1, although *C. pomonella* often prevailed, with the only exception at the Erasmo monitoring site in 2024. In contrast, significant differences emerged more consistently from period 2 onwards, and especially during the late ripening period and after husk dehiscence (Periods 3 and 4), when *E. ceratoniae* was usually dominant, with just a few site‐ and year‐specific exceptions (Erasmo 2023 and Mandrile 2024), where differences were not significant. Taken together, Tables 4 and 5 show that capture differences between species were generally not significant during Period 1, whereas significant differences were more frequently detected from Period 2 onwards and in Periods 3 and 4.

### 3.4 Integrated analysis of phenology and pest dynamics

**Figure 4:**
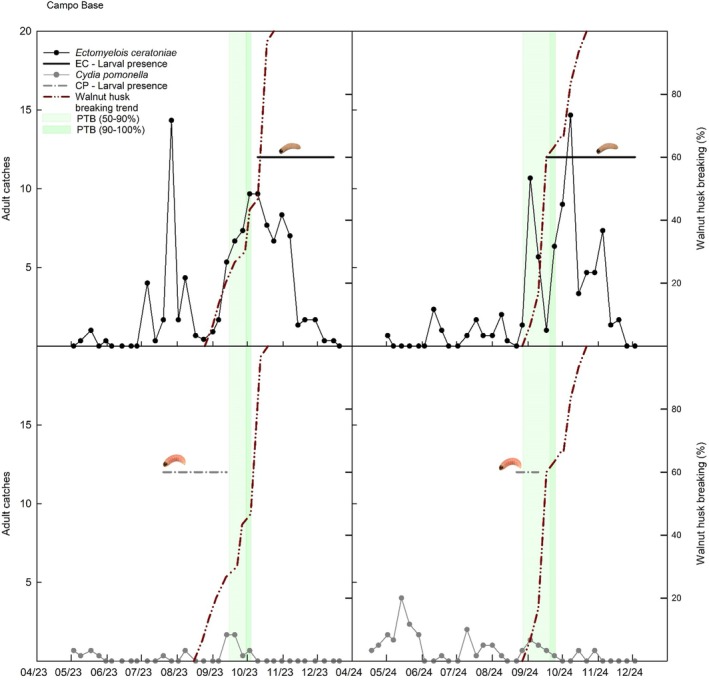
Integrated phenological and insect data for Campo Base in 2023 and 2024. Upper panels refer to *Ectomyelois ceratoniae* and the lower panels to *Cydia pomonella* adults. Panels show weekly adult captures, periods of larval presence in walnuts, and the percentage of husk dehiscence and PTB stages (50–90% and 90–100%).

**Figure 5:**
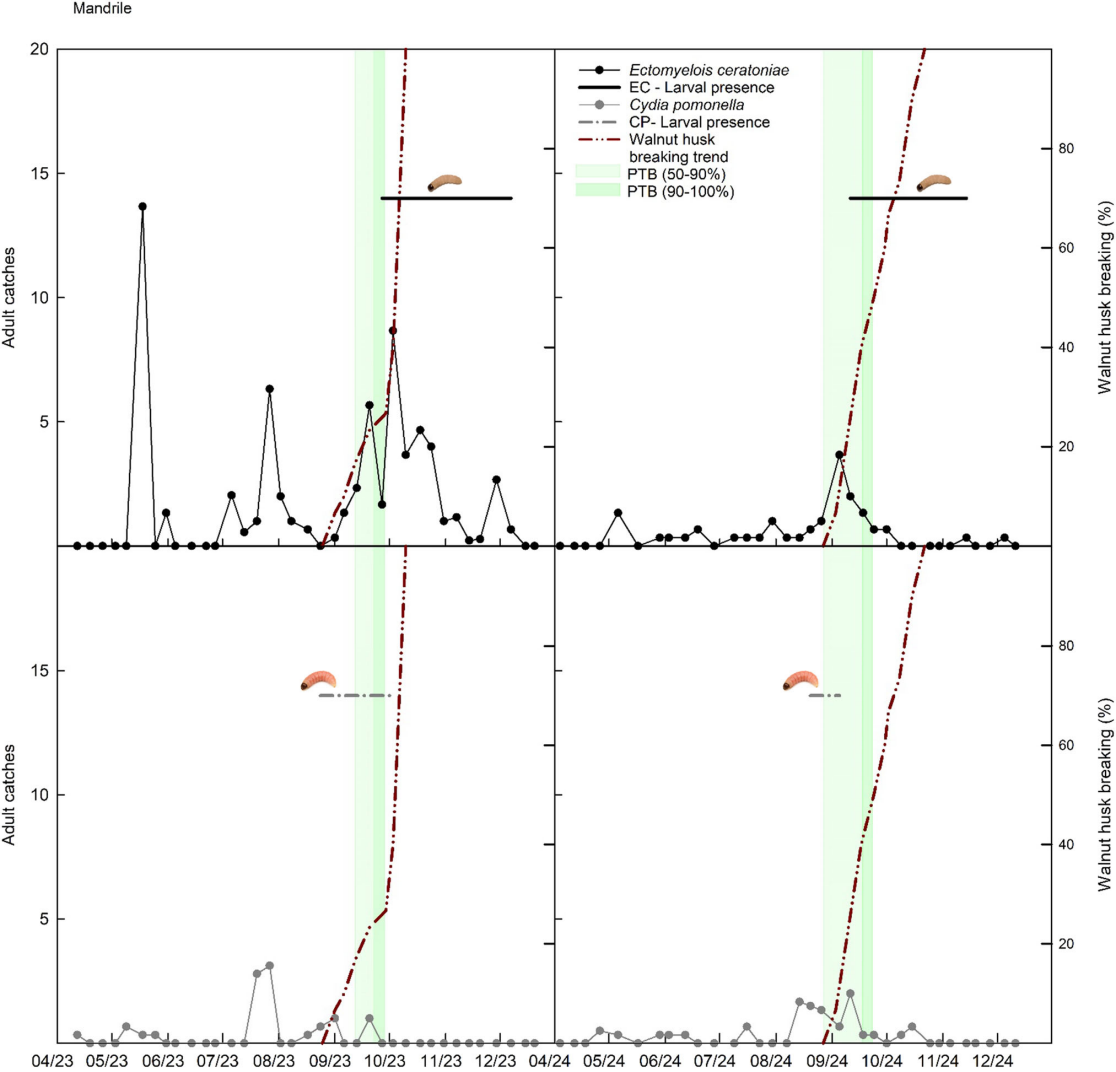
Integrated phenological and insect data for Mandrile in 2023 and 2024. The upper panels refer to *Ectomyelois ceratoniae* and the lower panels to *Cydia pomonella* adults. Panels show weekly adult captures, periods of larval presence in walnuts, and the percentage of husk dehiscence and PTB stages (50–90% and 90–100%).

**Figure 6:**
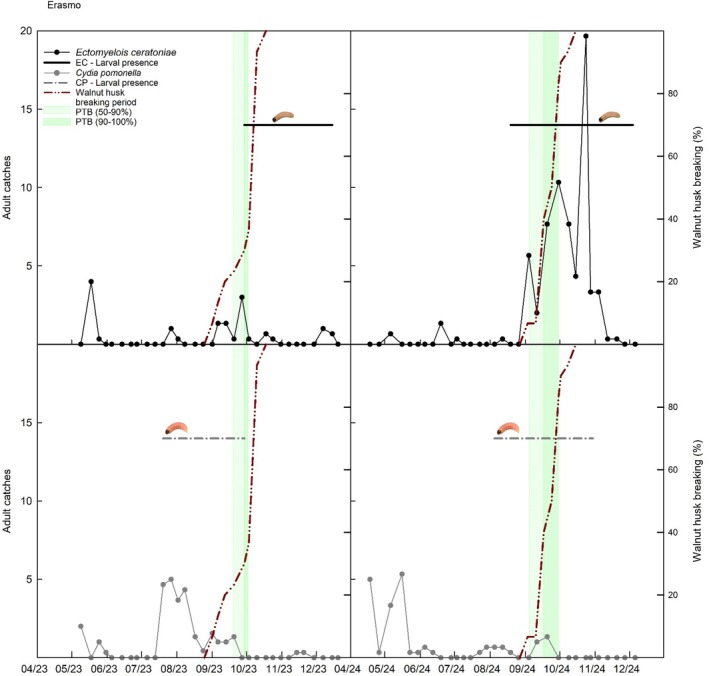
Integrated phenological and insect data for Erasmo in 2023 and 2024. The upper panels refer to *Ectomyelois ceratoniae* and the lower panels to *Cydia pomonella* adults. Panels show weekly adult captures, periods of larval presence in walnuts, and the percentage of husk dehiscence and PTB stages (50–90% and 90–100%).

Phenological progression, adult moth captures, and larval presence were analysed together within a phenology‐based framework to investigate the temporal relationships between walnut fruit development stages and pest activity. Figures 4, 5, 6 show the integrated trends of adult trap catches, onset of larval presence within fruits, husk breaking, and PTB for Campo Base, Erasmo, and Mandrile, respectively.

Larval infestation by *E. ceratoniae* coincided with advanced walnut ripening, becoming evident only after husk dehiscence and usually at the PTB stage. Conversely, *C. pomonella* larvae were detected earlier, sometimes before either husk dehiscence or the PTB stage. Adult capture dynamics reflected these differences, with *E. ceratoniae* showing pronounced flight peaks overlapping with husk dehiscence, while *C. pomonella* catches were comparatively sparse. These temporal associations were further examined through statistical comparisons of trap captures across phenological periods (Tables 4 and 5).

## 4 DISCUSSION

Walnut ripening phenology plays a central role in determining the vulnerability of fruits to infestation. Kernel development proceeds through well‐defined physiological stages, including the PTB stage, husk dehiscence, and hull maturity.^30^ These stages not only influence fruit quality and harvest timing but also condition susceptibility to lepidopteran pests. Observations indicate that *E. ceratoniae* preferentially attacks fruits at advanced ripening stages, when spontaneous dehulling and tissue softening facilitate oviposition and larval penetration. Synchrony between pest development and fruit phenology therefore determines the infestation pressure.

### 4.1 Walnut phenology

Phenological observations indicated interannual differences in PTB timing, with an earlier occurrence in 2024 compared to 2023. This confirms that walnut phenology can vary markedly across years and sites, potentially influencing its synchrony with pest activity. Despite this variability, the interval between pistillate flower receptivity and the PTB stage remained highly stable across years and sites (126–129 days), indicating that this phenological marker can be a useful way to estimate risk. This relationship may provide growers with a practical tool to anticipate harvest timing and to plan preventive interventions against *E. ceratoniae*.

The PTB stage proved to be a reliable indicator of kernel physiological maturity and a critical benchmark for balancing nut quality and pest risk. Previous studies have shown that kernels harvested at or shortly after PTB display optimal quality,^35^, ^36^ whereas slightly earlier harvests (pre‐PTB) may further enhance specific quality traits^28^ and help reduce carob moth infestation by removing fruits before they become highly susceptible to oviposition and larval penetration.

Overall, these findings underline the value of PTB as a phenological reference for timing harvest and pest management actions in walnut orchards. Phenological observations were used as a comparative and temporal framework rather than as a response variable for formal modelling, with the aim of identifying stable markers relevant for pest risk assessment.

### 4.2 Lepidoptera infestation and kernel infestation incidence

In this study, infestation was quantified as the seasonal incidence of larvae within the kernel, and no direct assessment of yield loss or quality. Postharvest outcomes were not evaluated, and therefore no inference can be made on the persistence of infestation at harvest or during storage. The results are intended to inform field‐level risk assessment and the timing of preventive measures rather than postharvest damage. Statistical comparisons showed that the total number of captures was significantly higher for *E. ceratoniae* than for *C. pomonella* in all orchard‐year combinations, with the exception of Erasmo in 2023 and Mandrile in 2024. These site‐ and year‐specific deviations likely reflect local ecological conditions, including interannual variability in phenological synchrony and differences in the availability of alternative hosts in the surrounding landscape. Even in these exceptions, *E. ceratoniae* remained a major component of the lepidopteran complex, reinforcing its predominance in Mediterranean walnut orchards.

Larvae of *E. ceratoniae* were mainly recovered at advanced ripening stages, coinciding with husk dehiscence and softening of husk tissues, which facilitate oviposition and direct kernel penetration. Similar dynamics have been reported on pomegranate and pistachio, where infestation levels and demographic performance were strongly influenced by fruit developmental stage.^15^, ^19^, ^22^ On almonds, oviposition likewise occurs mainly after hull split, as females lay eggs within hull‐opening cracks, which later facilitate larval entry.^37^ This suggests that *E. ceratoniae* tends to avoid early husk development and exploits late phenological stages to reach the kernel more directly.

The results also indicate that *C. pomonella* was present in the orchards, although always at lower levels during the monitoring period. The very low number of larvae recovered from walnuts suggests that this species probably prefers to oviposit on alternative hosts, particularly apple, which are abundant in the surrounding landscape.

In addition to the differences in abundance, the two species exhibited distinct temporal and developmental patterns. The transition from husk colonization to kernel penetration occurred earlier for *C. pomonella*, whose larvae were typically observed first in the husk and only later in the kernel. In contrast, *E. ceratoniae* larvae were frequently recovered directly in the kernel, confirming a strong association with fully or partially dehiscent fruits. These observations indicate that control measures targeting *C. pomonella* should be scheduled earlier in the season, whereas those against *E. ceratoniae* can be more effectively timed according to walnut phenology. The comparison thus highlights a partial ecological overlap, between the two species but the higher incidence of *E. ceratoniae* at late walnut stages, combined with its direct kernel colonization, clearly identifies it as the primary lepidopteran pest in Mediterranean walnut systems.

### 4.3 Lepidoptera adult monitoring with traps

Trap data confirmed the predominance of *E. ceratoniae*, with interannual and site‐specific fluctuations. Early‐season differences were generally negligible (Period 1), when *C. pomonella* often prevailed. Whereas from Period 2 onwards, and especially during late ripening and post‐husk dehiscence (Periods 3 and 4), *E. ceratoniae* usually dominated, with few site–year exceptions (Mandrile 2024; Erasmo 2023), a pattern that is interpreted primarily in relation to phenology‐driven temporal dynamics despite the possibility of species‐specific differences in pheromone lure attractiveness. These fluctuations may be related to the carob moth's polyphagy and to the variable availability of host plants across seasons and landscapes. Mean weekly captures (number of adults per trap per week) reached the highest values in Period 4 at Campo Base (44.7 in 2023; 46.7 in 2024), confirming the strong association with late ripening.

Overall, these patterns reinforce the dominant role of *E. ceratoniae* in Mediterranean walnut orchards, particularly during late ripening.

Adult captures preceded larval infestation primarily during the late‐season susceptible window, when walnut fruits become accessible and suitable for larval development, likely representing a protected resource exploited late in the season by a highly polyphagous species. Under this phenology‐driven context, pheromone traps provide an early warning of infestation risk when interpreted in relation to walnut phenology. The formulation lacking butylated hydroxytoluene did not produce reliable captures, whereas the stabilized lure based on 7*Z*,9*E*,11‐dodecatrienyl formate used in 2023 and 2024 consistently yielded measurable flight curves and allowed accurate monitoring of male activity. This role is consistent with observations in other crops such as date palm, where pheromone‐based monitoring has been used to support decision‐making and define action thresholds.^38^ In tree nut orchards, pheromone‐based monitoring has become essential for timing management interventions, as shown for navel orangeworm in California, where integration of trapping data with crop phenology underpins control decisions.^39^, ^40^

### 4.4 Integrated analysis of phenology and pest dynamics

This section integrates phenological observations, adult flight dynamics, and larval infestation patterns to provide a unified interpretation of pest–host synchrony, rather than presenting additional independent results. The combined analysis of phenology, adult captures, and larval infestation demonstrated that *E. ceratoniae* pressure is closely associated with husk dehiscence and the PTB stage consistent with the highest captures recorded during Period 4, when male activity peaked in both years. In contrast, *C. pomonella* activity prevailed early in the season, with larvae detected even before husk dehiscence and PTB stage. This confirms that the two species exploit different temporal windows of walnut susceptibility. *Cydia pomonella* is linked to early fruit development, whereas *E. ceratoniae* predominates during late ripening. The transition from husk colonization to kernel penetration was also species‐specific, with *C. pomonella* larvae typically moving from husk to kernel, while *E. ceratoniae* larvae were most often recovered directly in the kernel, reflecting oviposition preference for partially or fully dehiscent fruits. A recent study in Türkiye consistently reported the same temporal separation, with codling moth dominating early‐season infestations and carob moth late‐season infestations, both causing comparable economic losses.^25^ However, unlike the Turkish scenario Italian orchards showed a clear predominance of *E. ceratoniae* throughout the susceptible period, while *C. pomonella* remained consistently marginal. These discrepancies may reflect differences in cultivar composition, local climate, and the availability of alternative hosts in the surrounding landscape.

Consequently, walnut susceptibility to *E. ceratoniae* peaks during late ripening, coinciding with pronounced kernel compositional and quality changes.^28^

Although the PTB stage is primarily an agronomic threshold used to define the optimal harvest window for nut quality, it also coincided with peak infestation pressure, thus providing a practical reference point for aligning pest management with crop maturity. At this stage, infestations compromise both yield and nut quality, resulting in cumulative economic losses. Postharvest studies have similarly shown that later‐harvested walnuts not only suffer greater insect damage but also exhibit increased rancidity and poorer kernel appearance, highlighting PTB stage as a critical threshold for both pest management and nut quality.^36^

A phenology‐based framework is therefore essential, integrating flight curves, larval monitoring, and fruit maturity indicators to improve the timing of interventions within IPM strategies. Similar patterns have been reported for *Amyelois transitella* in California, where late‐season fruits at husk dehiscence are highly susceptible, and synchrony between kernel maturity and oviposition determines damage intensity.^39^, ^40^ In California, control of *A. transitella* in walnut and other nut crops is based on a combination of winter sanitation, mating disruption, insecticide applications timed with crop phenology, and early harvest. Among these measures, winter sanitation has shown the highest impact in reducing pest populations and supporting the effectiveness of other interventions.^39^ These approaches can serve as a useful reference for developing similar phenology‐driven strategies against *E. ceratoniae* in Mediterranean orchards.

Early studies also showed that delaying harvest beyond PTB stage significantly increased insect damage and reduced kernel value.^35^ These experiences may serve as a reference for Mediterranean walnut orchards, where *E. ceratoniae* predominates, while in California it is marginal compared with *A. transitella*. This difference may be linked to cultivar composition, ecological displacement, or the polyphagy of *E. ceratoniae*, which may develop on alternative hosts when walnuts are less suitable.

Persistence of larvae inside walnut fruits until late maturity was observed, mirroring pomegranate, where late‐season larvae entered diapause and overwintered in fruits, acting as a major source of next season infestations.^11^ In Iraq, orchards without sanitation reached > 90% infestation, *versus* values not exceeding 60% in orchards with sanitation.^11^ Beyond field infestations, larvae can persist and reproduce in stored nuts, causing continuous postharvest damage.^13^, ^23^, ^41^ This emphasizes the role of orchard sanitation, removal or destruction of infested fruits, as a critical IPM component, and support integrating sanitation and early harvest with monitoring and complementary measures.

Such measures have also been validated in citrus and other hosts, where mass trapping, *Trichogramma* releases, and mating disruption significantly reduced carob moth infestations.^12^ Moreover, fruit nutritional and biochemical traits can influence susceptibility to *E. ceratoniae*: in pomegranate, infestation levels were negatively correlated with peel hardness, calcium (Ca), potassium (K), silicon (Si) concentrations, juice acidity, and total phenolic content, while softer and sweeter fruits were more heavily attacked.^42^ Although such relationships remain unexplored in walnut, they suggest that susceptibility evaluations should also include crop quality traits into pest risk assessment.

Finally, it is worth noting that regional integrated production guidelines in Italy, and likely in other European countries, still do not list *E. ceratoniae* among walnut pests. As a result, no dedicated treatments are foreseen, and the threat posed by this species may be systematically underestimated.

## 5 CONCLUSION AND PROSPECTS FOR IPM

The results clearly demonstrate the predominance of *E. ceratoniae* over *C. pomonella* across sites and years, corroborating its role as the primary lepidopteran pest of late‐season walnuts in Mediterranean orchards. Pest pressure was tightly linked to fruit ripening, particularly the PTB stage and husk dehiscence, while pheromone trap captures consistently preceded larval entry, validating traps as effective early‐warning tools. Early harvest immediately after PTB stage, combined with orchard sanitation and complementary IPM tactics, can substantially reduce infestation risk. This study identifies key phenological stages of walnut susceptibility, quantifies *E. ceratoniae* infestation across these stages, and highlights their relevance for predicting and mitigating late‐season infestation risk. Overall, the findings provide a practical, phenology‐driven IPM framework to safeguard walnut quality and support the inclusion of *E. ceratoniae* in official production guidelines for Mediterranean walnut systems.

## FUNDING INFORMATION

This work was supported by the Campania Region Phytosanitary Office under the URCoFi (Unità Regionale di Coordinamento Fitosanitario) project.

## AUTHOR CONTRIBUTIONS

UB conceived and supervised the study, coordinated project administration, and led the writing of the original draft and its revision. FB and FMiele contributed equally to experimental design and field investigations, with FB leading data analysis and manuscript preparation. FMiele, EP, GA, and FMigliaccio participated in field monitoring and laboratory work. FN and EP contributed to data interpretation and critical manuscript revision. All authors read and approved the final version and agree to be accountable for all aspects of the work.

## Data Availability

The data that support the findings of this study are available from the corresponding author at francesco.nugnes@cnr.it upon reasonable request.
